# The phenotypic plasticity of developmental modules

**DOI:** 10.1186/s13227-016-0053-7

**Published:** 2016-08-02

**Authors:** Aabha I. Sharma, Katherine O. Yanes, Luyang Jin, Sarah L. Garvey, Sartu M. Taha, Yuichiro Suzuki

**Affiliations:** 1 Department of Biological Sciences, Wellesley College, 106 Central St., Wellesley, MA 02481 USA; 2 Departments of Pathology and Microbiology-Immunology, Northwestern University Feinberg School of Medicine, Ward Building 4-075, 303 East Chicago Avenue, Chicago, IL 60611 USA; 3 The University of Texas Health Science Center at San Antonio, 7703 Floyd Curl Dr., San Antonio, TX 78229 USA

**Keywords:** Phenotypic plasticity, Robustness, Wnt signaling, *Oncopeltus fasciatus*, Melanin, Regeneration, Pigmentation

## Abstract

**Background:**

Organisms develop and evolve in a modular fashion, but how individual modules interact with the environment remains poorly understood. Phenotypically plastic traits are often under selection, and studies are needed to address how traits respond to the environment in a modular fashion. In this study, tissue-specific plasticity of melanic spots was examined in the large milkweed bug, *Oncopeltus fasciatus*.

**Results:**

Although the size of the abdominal melanic bands varied according to rearing temperatures, wing melanic bands were more robust. To explore the regulation of abdominal pigmentation plasticity, candidate genes involved in abdominal melanic spot patterning and biosynthesis of melanin were analyzed. While the knockdown of *dopa decarboxylase* (*Ddc*) led to lighter pigmentation in both the wings and the abdomen, the shape of the melanic elements remained unaffected. Although the knockdown of *Abdominal-B* (*Abd-B*) partially phenocopied the low-temperature phenotype, the abdominal bands were still sensitive to temperature shifts. These observations suggest that regulators downstream of Abd-B but upstream of DDC are responsible for the temperature response of the abdomen. Ablation of wings led to the regeneration of a smaller wing with reduced melanic bands that were shifted proximally. In addition, the knockdown of the Wnt signaling nuclear effector genes, *armadillo1* and *armadillo 2*, altered both the melanic bands and the wing shape. Thus, the pleiotropic effects of Wnt signaling may constrain the amount of plasticity in wing melanic bands.

**Conclusions:**

We propose that when traits are regulated by distinct pre-patterning mechanisms, they can respond to the environment in a modular fashion, whereas when the environment impacts developmental regulators that are shared between different modules, phenotypic plasticity can manifest as a developmentally integrated system.

**Electronic supplementary material:**

The online version of this article (doi:10.1186/s13227-016-0053-7) contains supplementary material, which is available to authorized users.

## Background

Many organisms develop and evolve in a modular fashion [1–4]. The role of genes in shaping developmental modules, or traits that are tightly linked by strong interactions, has been extensively studied, but how these modules respond to different environments remains poorly understood. Phenotypic plasticity is the ability of a given genotype to produce variable phenotypes in different environments [5, 6]. Although many traits exhibit phenotypic plasticity [7–9], little is known about phenotypic plasticity in the context of modularity. Understanding how trait-specific plasticity arises is particularly important in systems that develop and evolve in a modular fashion and should provide insight into evolvability, the ability of an organism to evolve.

Melanization offers an excellent opportunity to explore the mechanisms that give rise to tissue-specific differences in plasticity. Melanization is an ecologically important trait that provides various functions from thermoregulation and desiccation tolerance to sexual selection and crypsis [10, 11]. Whereas many vertebrates have robust melanization patterns that are genetically determined, melanization in invertebrates can be highly plastic [11]. Melanic elements, such as spots and bands, share similar structural components, melanins, and develop using the same biochemical processes. However, their degree of plasticity can vary depending on the location of the body. Thus, disrupting the melanin biosynthesis biochemical pathway can lead to seemingly coordinated changes in these melanic elements, but individual elements can often differ in their plasticity and patterning mechanisms, indicating that they are in fact distinct modules. We sought to investigate how environmental changes impact a system where distinct modules share common developmental pathways.

The cellular and molecular mechanisms underlying cuticular melanization have been studied extensively in various insects, such as the fruit fly, *Drosophila melanogaster*, and the tobacco hornworm, *Manduca sexta* [12–18]. The extensive literature on the developmental regulation and biochemical synthesis of melanins provides a unique opportunity to study how phenotypic plasticity is regulated in a tissue-specific manner. Furthermore, melanic patterns are two-dimensional structures that are relatively simple to analyze.

Two distinct processes regulate the production of melanic elements: the biochemical synthesis of melanin and the patterning of the melanic elements. The biosynthesis of melanin involves a series of enzymatic steps that ultimately convert tyrosine to dopa melanin and dopamine melanin [13, 18, 19]. Plasticity in the expression of melanin synthesis enzymes, such as dopa decarboxylase (DDC), has been shown to regulate plasticity in pigmentation [20].

In addition to biochemical synthesis, melanic elements are often spatially restricted to particular portions of the body by patterning mechanisms. Recent studies have highlighted the importance of transcription factors and signaling molecules in regulating the location, size and shape of pigmentation [15, 21–24]. In the present study, we focus on melanic patterns found on the abdomen and the wings. In the abdomen of *Drosophila*, the posterior Hox gene, *Abdominal*-*B* (*Abd*-*B*), regulates sex-specific melanization patterns [12, 18] and mediates their phenotypic plasticity [12]. In the wings, Wnt signaling has recently been shown to regulate the development of pigmentation in *Drosophila* and several lepidopteran species [25–28]. There are several different Wnt ligands, which, in the canonical Wnt signaling pathway, bind to the cell surface receptor Frizzled and trigger the cytoplasmic stabilization and nuclear localization of Armadillo (Arm) [29, 30]. Arm in turn binds to the DNA-binding protein Tcf, converting it from a transcriptional repressor to a transcriptional activator of downstream genes [29]. It has been known for some time that the loss of one of the Wnt genes, *wingless* (*wg*), results in the transformation of wings into the notum in *Drosophila* and that Wg promotes growth of wings [31, 32]. However, Wnt signaling recently was also shown to be necessary for generating the melanic spots on *Drosophila* wings [33, 34]. In Lepidoptera, the expression patterns of several different Wnt ligands have been shown to correlate with the symmetry systems of the nymphalid ground plan [25–28], the idealized relationships between various spots and band in butterflies. Therefore, Wnt signaling appears to play an important role in growth and patterning as well as pigmentation of insect wings.

The milkweed bug *Oncopeltus fasciatus* (Heteroptera) is an emerging model species that has a sequenced genome and is amenable to functional analyses of genes using RNA interference [35]. The abdominal melanic bands of *Oncopeltus* are affected dramatically by temperature [36], but not all melanic elements are affected by temperature to the same extent. Thus, melanization in *Oncopeltus* provides a useful opportunity to begin deciphering the mechanisms underlying tissue-specific phenotypic plasticity. Melanization of both the abdomen and the wings in this species is regulated by the same set of enzymes involved in melanin production, such as DDC [37]. Thus, we hypothesized that the expressions of melanization enzymes must be altered in a tissue-specific manner for the melanic patterns to exhibit distinct degrees of phenotypic plasticity. Specifically, we focused on the melanic elements of the abdomen and the wings and explored the distinct mechanisms underlying their development.

## Methods

### Animals

Wild-type milkweed bugs, *Oncopeltus*, were obtained from Carolina Biological and raised in plastic containers on organic sunflower seed and water at 26.5 °C. For the temperature experiments, the milkweed bugs were raised separately at 20, 26.5 and 33 °C. The photoperiod was 16 h light:8 h dark.

### Sensitive period determination

The final (fifth) instar intermolt period of the *Oncopeltus* colony in our laboratory typically lasts at least 19 days at 20 °C and 6 days at 33 °C. To determine the temperature-sensitive period of pigmentation, different individuals raised at 20 °C were transferred to 33 °C on every other day of their fifth nymphal instar (day 0 through day 18). Similarly, different individual nymphs raised at 33 °C were transferred to 20 °C daily from day 0 through day 5 of the fifth instar. The nymphs were transferred every day given the short duration of the fifth instar when raised at 33 °C.

### Imaging and quantification of pigmentation

Whole bodies of *Oncopeltus* were fixed in 3.7 % formaldehyde and stored in 80 % glycerol at −20 °C for up to 1 week. The wings and ventral abdomen of each insect were mounted in 80 % glycerol and imaged using Nikon SMZ 1500 Microscope with 18.2 Color Mosaic Diagnostic instruments Insight Firewire Spot 2 Megasample camera. The area of melanic pigmentation was analyzed using ImageJ (NIH). The area of the abdominal melanic bands was normalized to body size by dividing the area by the total area of the A2–A4 abdominal sternites. In this study, only the forewing was studied. In *Oncopeltus*, the forewings have two melanized areas: the proximal melanic band and the distal membranous wing, which is also melanized. The distal membranous portion is structurally distinct, whereas the proximal band, similar to the abdominal pigmentation, is not associated with a structurally distinct element. The areas of the melanized portions were normalized to wing size by dividing the melanized area by the total area of the wing. All statistical analyses were performed using JMP Pro 9.

### Wing ablation

To investigate the melanic band formation when wings were ablated, the right forewings of the third, fourth and fifth instar *Oncopeltus* were ablated. Nymphs were anesthetized on ice and placed on a double-sided tape, and the wings pads were ablated using microscissors under a dissection microscope.

### mRNA isolation and PCR

Fifth instar *Oncopeltus* nymphs were dissected in 1X phosphate-buffered saline (0.02 M PBS; 0.15 M NaCl, 0.0038 M NaH_2_PO_4_, 0.0162 M Na_2_HPO_4_; pH 7.4). The gut and fat body were removed, and the remaining tissue was homogenized in Trizol^®^ (Life Technologies). Chloroform was added to the homogenized sample, and the supernatant containing RNA was extracted and precipitated in isopropanol. The pellet was washed with 75 % ethanol and resuspended in DEPC water. The isolated RNA was digested with DNase (Promega) and precipitated in isopropanol. cDNA was synthesized using the cDNA synthesis kit (Fermentas) following the manufacturer’s instructions.

### Cloning and double-stranded RNA (dsRNA) synthesis

The sequences for *Ddc* (GenBank: KM247781), *abdominal*-*A* (*abd*-*A*) (GenBank: FJ851728), *Abd*-*B* (GenBank: AY627362), *arm1* and *arm2* (Additional file 1) were amplified through PCR using primers listed in Table 1. The two *armadillo* genes, *arm1* and *arm2*, were identified in the *Oncopeltus* genome [38]. The PCR products were inserted into a TOPO-TA vector (Life Technologies), and TOP10 chemically competent bacterial cells were transformed with these vectors. The identity of the plasmid was confirmed via sequencing. The plasmid DNA was linearized via restriction enzyme digestion. Each of the dsRNA strands was synthesized using T3 and T7 MEGAscript kits (Life Technologies) following the manufacturer’s instructions. Equal amounts of single-stranded RNAs were hybridized to form 2 µg/µL solution of dsRNA in DEPC water [35]. The annealed dsRNA product was analyzed via agarose gel electrophoresis for confirmation of proper annealing.

**Table 1:** Primer sequences for various genes

Gene	dsRNA preparation primer sequence	
*arm1*	FW: 5′-AAGATGGTCTCCTTGCTTCA-3′	
	RV: 5′-AATCGCTGGTTTGTTGCTC-3′	
*arm2*	FW: 5′-AGTAAAATGGCTGTGCGTGT-3′	
	RV: 5′-CCCTGAGAGGCAAGAATGA-3′	
*abd-A*	FW: 5′-AGGGCGGTGAAGGAGATAA-3′	
	RV: 5′-TCTGGTGGTGCTGTTGGT-3′	
*Abd-B*	FW: 5′-GCCAACAACAACAACAGCA-3′	
	RV: 5′-GGTGTTTCATGGCTCCAC-3′	
*Ddc*	FW: 5′-CACAGAGCTGGAAGTGGTGA-3′	
	RV: 5′-CCATTCTGGGTGTTCTGCTT-3′	

Gene	Knock down verification primer sequence	# of cycles used
*arm1*	FW: 5′-AGTCCGTGCTGTTCTACGC-3′	35
	RV: 5′-AGGGTCCAGAGGCAGTTCT-3′	
*arm2*	FW: 5′-TCATCGTCAAGGGTTGCT-3′	30
	RV: 5′-TCTGAACTAATCGCTGTGAAGG-3′	
*abd-A*	FW: 5′-CGGCTCAGTTCTACCACCA-3′	33
	RV: 5′-TTCTGGGGCTGTTCCATT-3′	
*Abd-B*	FW: 5′-GAGTTCCTCTTCAACGCCTAC-3′	33
	RV: 5′-CTGCGGTTTTGGTTCTTCT-3′	
*Ddc*	FW: 5′-TCCCGACAGCAAACTCCT-3′	37
	RV: 5′-TTCAGGTAGAGAGGGTCAACA-3′	
*rps3*	FW: 5′-TTGATACCCAAAACCCCTTG-3′	23/25/27
	RV: 5′-CAACCCCATACACTTGACCT-3′	

### dsRNA injection

Day 0 *Oncopeltus* fifth instar nymphs were anesthetized on ice and injected into their dorsal abdomen with 0.1 or 1 µg of *abd*-*A* or *Abd*-*B* dsRNA, respectively, using a 10-µL glass capillary needle connected to a syringe. For *Ddc* knockdowns, 0.05 µg of dsRNA was injected into day 0 fourth instar nymphs. For *arm1* and *arm2* knockdowns, 1 µg and 2 ng–1 µg of dsRNA, respectively, was injected into randomly selected fourth instar nymphs. Controls were injected with 1 µg of bacterial *ampicillinresistance* (*amp*^*r*^) dsRNA. Unless otherwise noted, all knockdown insects were raised at 26.5 °C.

### Knockdown verification

Semiquantitative reverse transcriptase polymerase chain reaction was performed in order to confirm knockdown of dsRNA-injected animals (Additional file 2). *Ribosomal protein subunit 3* (*rps3*) was used as a control for loading. Randomly selected fourth instar nymphs were injected with 1 µg of dsRNA for *amp*^*r*^, *abd*-*A*, *Abd*-*B*, *Ddc* or *arm1* dsRNA. For *arm2* knockdown verification, 10 ng of *arm2* dsRNA was injected. RNA was isolated from three whole bodies of day 2 fifth instar nymphs and converted to cDNA as described above.

### Morphometric analysis

Randomly selected fourth instar *Oncopeltus* were injected with 1 µg of *arm1* or *amp*^*r*^ dsRNA. Images of 12 *arm1* dsRNA- and 12 *amp*^*r*^ dsRNA-injected adult forewings were taken using a Spot camera attached to a microscope. Ten landmarks on the wing were digitized using ImageJ. All subsequent data analyses were performed using MorphoJ [39]. Briefly, a generalized Procrustes fit was used to superimpose the wings. A covariance matrix was then generated, and a principal component analysis was performed. In order to compare the shapes of *amp*^*r*^ and *arm1* knockdown wings, a discriminant function analysis was carried out on the Procrustes coordinates generated previously.

### Color analysis

To determine the darkness of the red color of the body, adults raised at 20 and 33 °C were imaged using a scanner. The mean gray value of the ventral side of the first abdominal segment was determined using ImageJ. This function converts an RGB image into a grayscale image and gives a mean gray value where darker values have lower numbers.

## Results

### Abdominal melanization exhibits higher temperature-dependent plasticity than forewing melanization

**Fig. 1:**
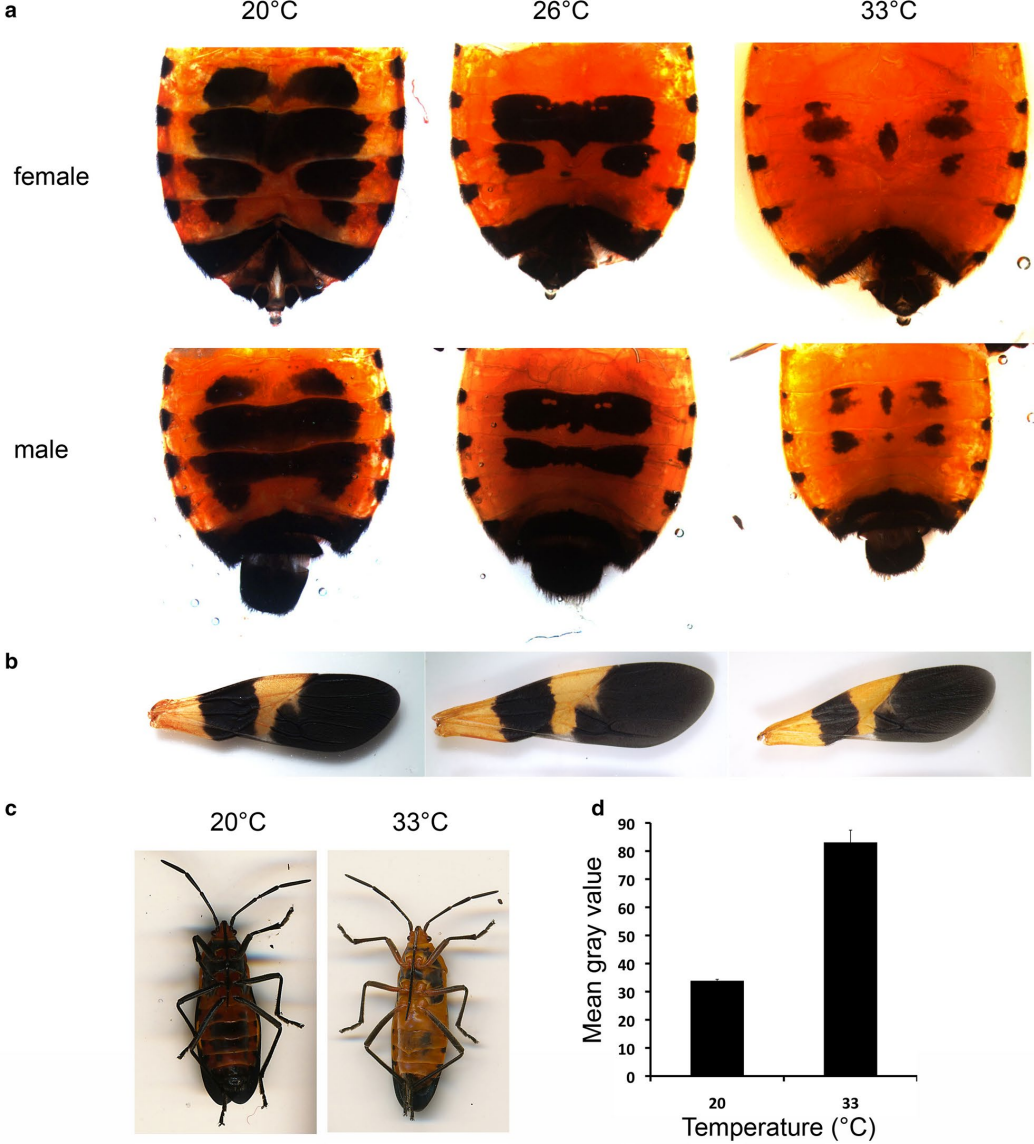
Effect of temperature on abdominal and wing pigmentation in male and female *Oncopeltus.* **a** Examples of ventral abdominal pigmentation of males and females reared at different temperatures. **b** Examples of forewing pigmentation of males and females reared at different temperatures. **c** Ventral side of an adult raised at 20 (*left*) and 33 °C (*right*). **d** Mean gray values of the ventral first abdominal segment of adults raised at 20 (*n* = 3) and 33 °C (*n* = 4). Student’s *t* test: *p* < 0.0001. *Error bars* represent standard error

**Fig. 2:**
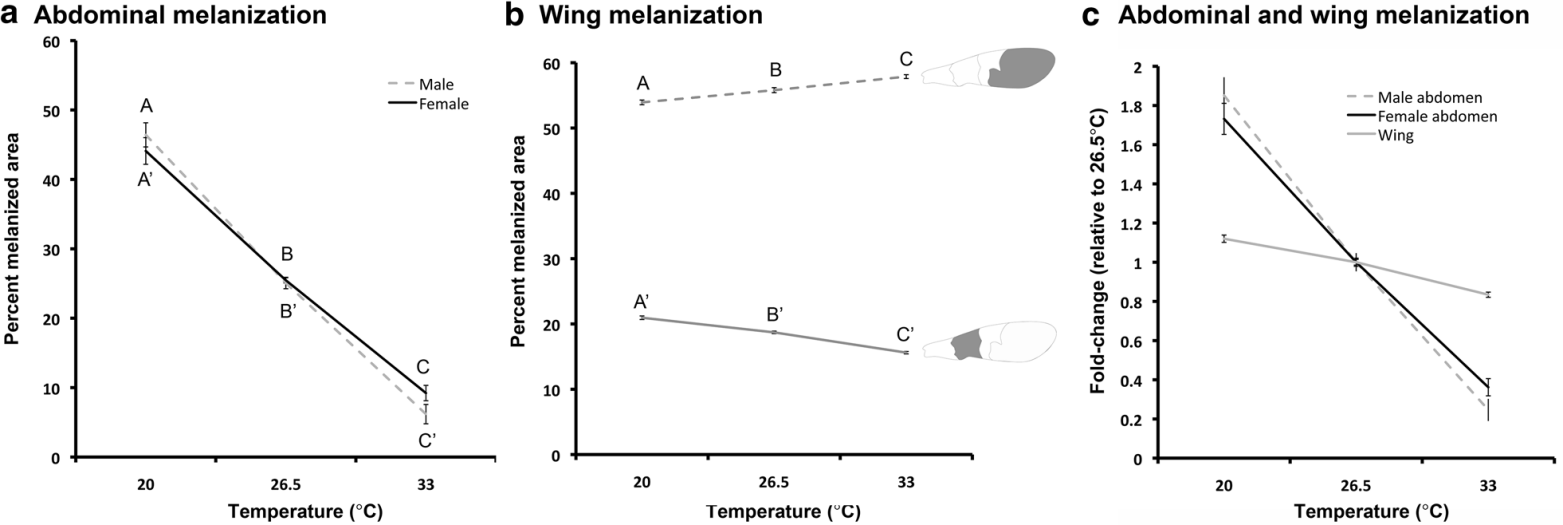
Reaction norms of abdominal and wing melanic pigmentation of males and females reared at different temperatures. **a** Reaction norm of abdominal melanic pigmentation. The area of melanic pigmentation was normalized for body size by dividing the area of the pigmented area by the total area of the A2–A4 abdominal segments. **b** Reaction norms of the proximal melanic band (*solid line*) and the distal melanized membranous portion of the wing (*dashed line*). A drawing of the forewing is shown with the measured areas highlighted in *black*. The areas were normalized for body size by dividing the pigmented area by the total wing area. In (**a**) and (**b**), the *error bars* represent standard error. Female: one-way ANOVA: *p* < 0.0001, *df* = 2, *F* = 189.42; Tukey–Kramer HSD analysis for females: *p* < 0.0001 for all pairwise comparisons. Male: one-way ANOVA: *p* < 0.0001, *df* = 2, *F* = 215.09; Tukey–Kramer HSD analysis for males: *p* < 0.0001 for all pairwise comparisons. **c** Fold difference of the normalized melanized areas of the abdomen and the proximal band of the wing relative to the normalized areas at 26.5 °C

We studied the ventral melanic abdominal pigmentation and wing melanization of adult *Oncopeltus* raised at three different temperatures, 20, 26.5 and 33 °C. Adults raised at a higher temperature had significantly less melanic abdominal pigmentation relative to those raised at lower temperatures (Fig. 1). The medial region of the abdomen appeared to be the most sensitive to temperature. The mean percent area of abdominal melanic pigmentation in both sexes of *Oncopeltus* raised at 20 °C was significantly larger than those raised at 26.5º and 33 °C (Fig. 2; female ANOVA: *p* < 0.0001, *df* = 2, *F* = 189.42; Tukey–Kramer HSD analysis for females: *p* < 0.0001 for all pairwise comparisons; male ANOVA: *p* < 0.0001, *df* = 2, *F* = 215.09; Tukey–Kramer HSD analysis for males: *p* < 0.0001 for all pairwise comparisons). The reaction norms for the abdominal melanization show that the percent melanized area of the abdomen varies with temperature in a continuous manner for the temperature range studied (Fig. 2).

Forewing melanization exhibited plasticity to a much lesser degree (Figs. 1, 2). We found that the normalized sizes of both the distal membranous portion of the forewing and the proximal band were relatively stable across all temperatures (Fig. 2). Interestingly, the normalized size of the distal melanized portion increased slightly at higher temperatures. Since the distal melanized portion corresponds to the membranous wing portion, we interpret this alteration to be caused by a shift in portion occupied by the membranous wing rather than a shift in melanization per se. In contrast, the proximal band showed a decrease in melanization with increasing temperatures. Although the percent melanized area of the proximal band showed significant changes in response to temperature changes (ANOVA: *p* < 0.0001, *df* = 2, *F* = 148.56), the fold difference in the melanized area between the animals raised at 20 and 33 °C was much smaller for the wings relative to that observed in the abdomen (Figs. 1, 2). The percent abdominal melanin areas differed by at least fourfold between 20 and 33 °C, but the percent normalized melanized area of the wing only changed by approximately 1.3-fold in the same temperature range. These results demonstrate that the wing melanization is much more robust than abdominal pigmentation.

In addition to the changes in the size of the black patterns, the redness of the body for those reared at 20 °C was significantly darker compared with those reared at 33 °C (Fig. 1c, d; *p* < 0.0001, Student’s *t* test). Thus, color plasticity was also seen in the whole body, not just the black melanic elements in the abdomen.

### The temperature-sensitive period for abdominal melanization

**Fig. 3:**
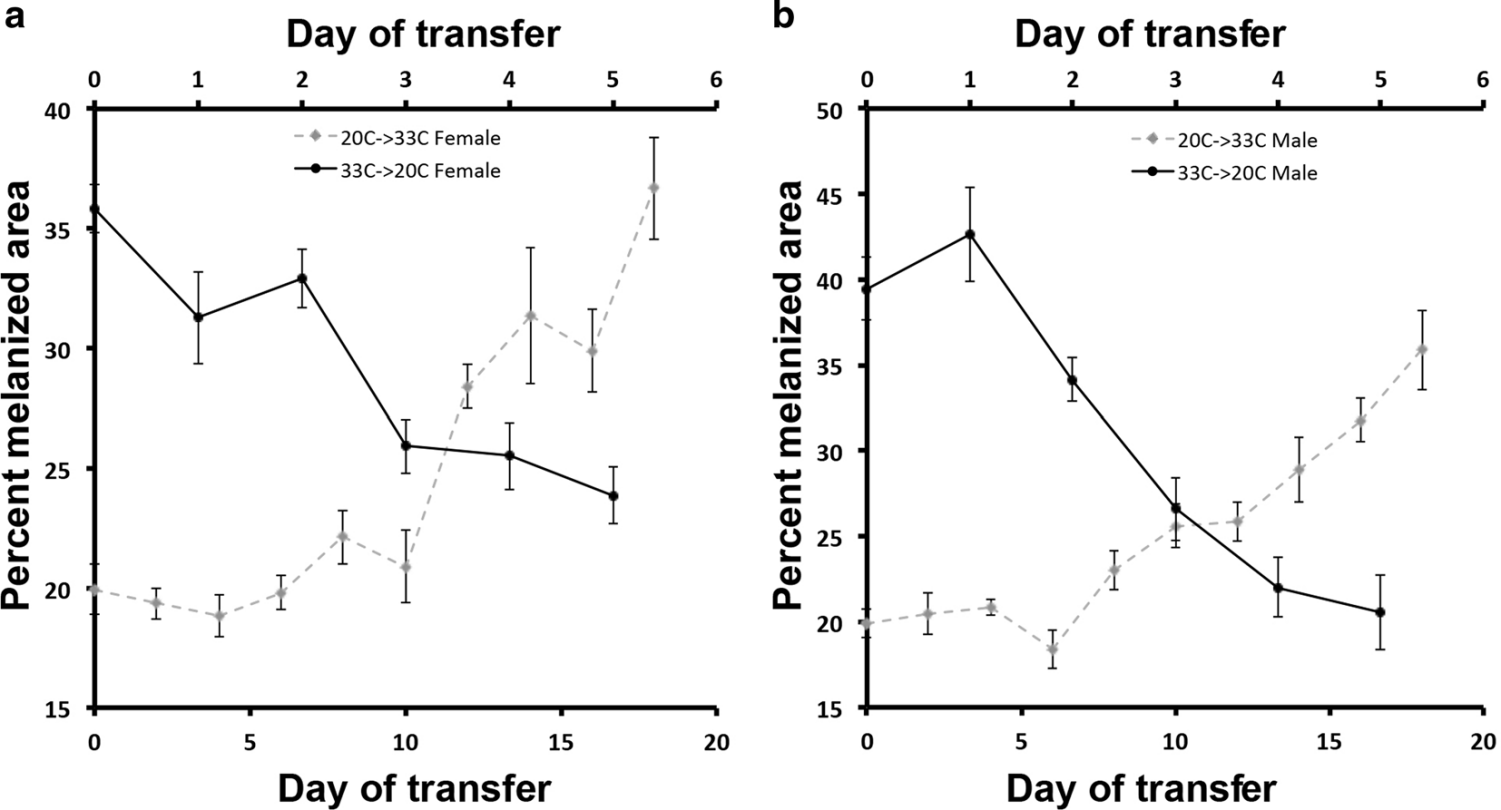
Temperature-sensitive period of the ventral melanic abdominal pigmentation during the fifth nymphal instar of *Oncopeltus*. The plot shows the average normalized melanic pigmentation of adult female (**a**) and male (**b**) raised at 20 or 33 °C and transferred to 33 (*dashed line*) or 20 °C (*solid line*), respectively, on various days. The *top X*-axis represents the day at 33 °C when the nymphs were transferred to 20 °C. The *bottom X*-axis represents the day at 20 °C when the nymphs were transferred to 33 °C. The melanized area was normalized by dividing the area of the pigmented area by the total area of the A2–A4 abdominal segments. Each data point represents average calculated from measurements of 5–13 animals

Next we aimed to determine when and how plasticity in the abdominal melanization arises by determining the sensitive period for the ventral abdominal melanic bands in fifth instar *Oncopeltus* raised at the extreme temperatures (20 and 33 °C). The *Oncopeltus* strain used in this study took 6 days to molt to an adult when raised at 33 °C, 7 days at 26.5 °C and 19–22 days at 20 °C from the onset of the final (fifth) nymphal instar. The fifth instar nymphs raised at 33 °C were transferred daily to 20 °C on day 0 through day 5, and fifth instar nymphs raised at 20 °C were transferred to 33 °C every other day of their fifth nymphal stage (day 0 through day 18). Based on the area of the ventral melanic abdominal bands of the transferred adults (Fig. 3), days 2–5 were found to be the sensitive period for 33 °C nymphs. No differences based on sex were observed for these adults. For 20 °C nymphs transferred to 33 °C, days 10–14 for males and 12–14 for females were found to be the most sensitive period for abdominal pigmentation (Fig. 3).

The observed phenotypic plasticity suggests a temperature-sensitive mechanism that determines the level of melanism before the adult cuticle is laid down. Our results suggest that the sensitive period for abdominal pigmentation occurs primarily during the mid-final nymphal stage of *Oncopeltus* although rearing conditions during the earlier nymphal stages and the final days of the last instar can have a minor impact on the final adult melanization (Additional file 3).

### Knockdown of Abd-B partially phenocopies low-temperature melanism but does not abolish temperature sensitivity

In *Drosophila*, abdominal melanization has been shown to be regulated partly by Abd-B [12]. Another study showed that the loss of the Hox gene *abd*-*A* leads to complete loss of melanism in segments A2–A8, possibly by the expansion of Abd-B mediated suppression of melanin production/deposition [40]. We have independently verified that the removal of *abd*-*A* leads to a complete loss of *Oncopeltus* ventral abdominal pigmentation (Additional file 4). We also observed melanization in the anterior and posterior margins of the abdominal sternites in adults with weaker knockdown effects. These effects are distinct from the phenotypic effects observed at high temperatures where melanin is conspicuously absent from the margins. Thus, *abd*-*A* knockdown does not appear to phenocopy the effects of temperature.

**Fig. 4:**
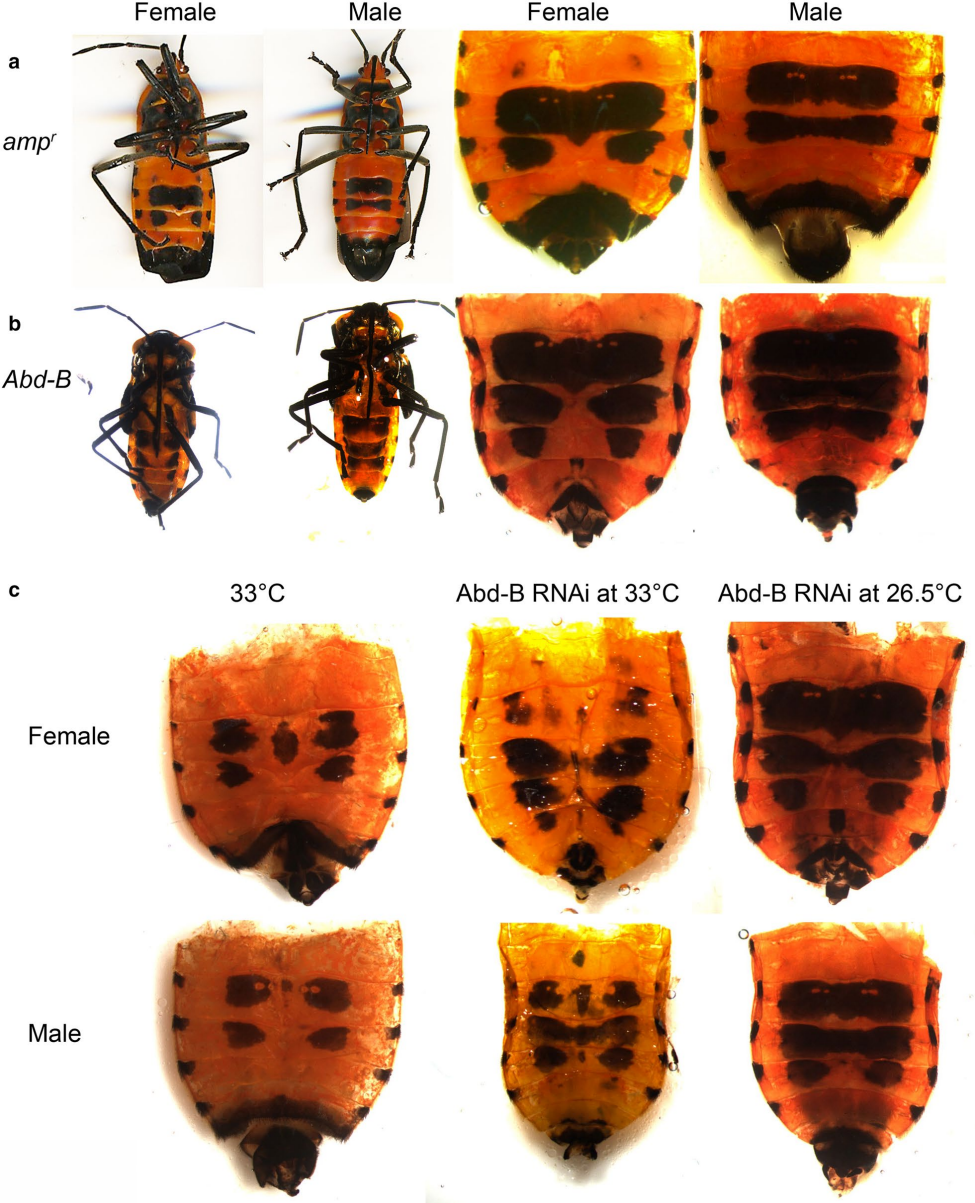
Effect of day 0 fifth nymphal stage *Abd*-*B* knockdown on the adult phenotype of *Oncopeltus.* **a**, **b** Full-body images (*left*) and ventral abdomens (*right*) of control *amp* ^*r*^ (1 µg) and *Abd*-*B* (1 µg) dsRNA-injected adult females and males raised at 26.5 °C. **c** Abdomens of *Abd*-*B* dsRNA-injected adult males and females raised at 33 °C. Abdomens isolated from wild-type adults raised at 33 °C and *Abd*-*B* dsRNA-injected adults raised at 26.5 °C are provided for comparison

Removal of *Abd*-*B* at 26.5 °C phenocopied phenotypes generated from raising the nymphs at 20 °C, resulting in extra melanic pigmentation in segment A5 that normally lacks pigmentation in the ventral abdomen of both male and female *Oncopeltus* (*n* = 29; Fig. 4). To determine whether the abdominal pigmentation retains sensitivity to temperature when *Abd*-*B* is knocked down*, Abd*-*B* was knocked down at 33 °C. Ectopic melanic pigmentation in the A5 segment persisted at 33 °C knockdown. However, the medial portions of each melanic pigment band disappeared (*n* = 8; Fig. 4c), suggesting that while *Abd*-*B* appears to play a role in specifying the segments where abdominal melanic bands develop, it is independent of the temperature-sensitive pathway involved in regulating the amount of melanin produced.

### Knockdown of *Ddc* leads to reduced darkness of the abdominal melanic bands without altering the shape

**Fig. 5:**
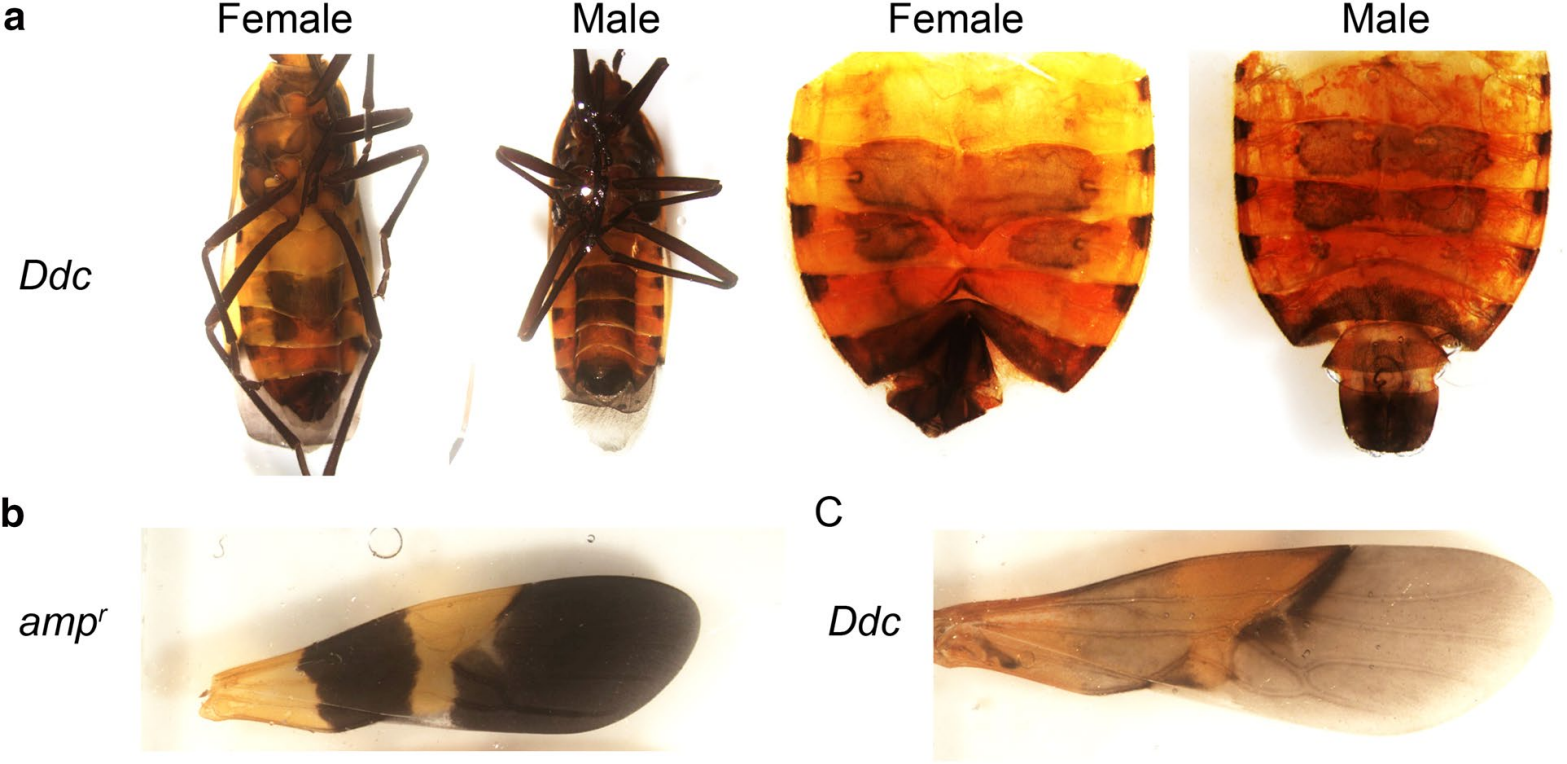
Effect of day 0 fifth nymphal stage *Ddc* knockdown on the adult phenotype of *Oncopeltus*. **a** Full-body images (*left*) and ventral abdomens (*right*) of *Ddc* (50 ng) dsRNA-injected adult females and males. **b** Wing from an *amp* ^*r*^ dsRNA-injected adult. **c** Wing from a *Ddc* dsRNA-injected adult

A recent study has shown that the removal of *Ddc* results in the complete loss of black coloration throughout the body [37]. Here, we examined the hypomorphic effects of *Ddc* knockdown. Injection of 50 ng of *Ddc* dsRNA resulted in the formation of faint abdominal bands (*n* = 13/18; Fig. 5). Although the melanization was reduced in intensity, the banding pattern of the abdomen looked similar to controls injected with *amp*^*r*^ dsRNA. Similarly, removal of DDC led to fainter wing melanic bands without affecting the overall shape of the melanic regions (Fig. 5b, c). Thus, *Ddc* regulates the amount of melanin deposited but not the size and shape of the melanic bands, which are under the control of temperature. Thus, temperature-specific plasticity in the abdominal melanic patterning must be regulated upstream of *Ddc*.

### Pattern regulation in regenerated wings

**Fig. 6:**
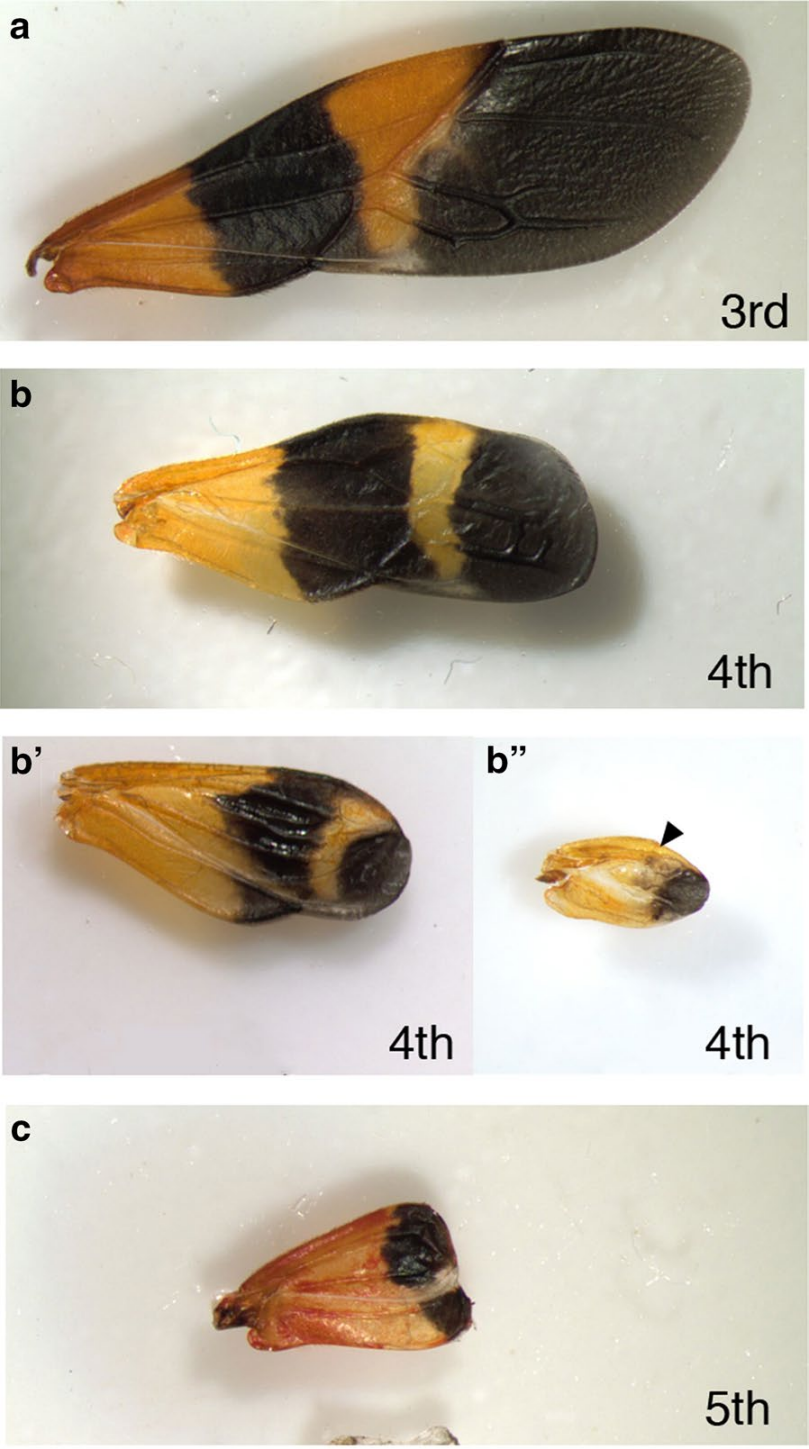
Effect of wing ablation on wing melanic patterns. **a**–**c** Effect of wing ablation on the melanic pattern of the adult wing. Wings were ablated on day 0 or 1 of the third (**a**), fourth (**b**–**b**″) or the fifth (**c**) instar nymphs. *Arrowhead* indicates the proximal black band that has shifted proximally

Next, we investigated the potential cause behind the robustness of melanic bands of the forewing. To determine how the melanic portions of wings might be established in *Oncopeltus*, wing pads of third, fourth and fifth instar nymphs were ablated. While removal of third instar wing pads led to successful regeneration during successive molts (*n* = 5 out of 6), ablation of wing pads of fourth instar nymphs resulted in much smaller adult wings (Fig. 6). The melanic elements were almost always present in all cases (Fig. 6). In these wings, the distal black membranous portion of the wing, medial orange area, and proximal black band of the adult wing were all reduced in size and shifted proximally (*n* = 9). In contrast, when wings of fifth instar nymphs were ablated, the wings failed to regenerate and all that formed were small orange wings or at most a small orange wing with a melanic area at the distal edge (*n* = 5). These nymphs most likely did not have enough time to regenerate before the adult wings formed. The reduction in wing size and the corresponding reduction in melanic band size as a result of fourth instar wing ablation were reminiscent of the phenotypes seen in regenerated nymphalid butterfly wings [41]. Because wing growth, marginal bands and symmetry system patterning appear to be co-regulated by the same signaling pathway [25, 42], one possible cause of the reduced plasticity in the *Oncopeltus* wing melanic bands may be the genetic costs of plasticity [43]. Such genetic costs arise when a pleiotropic factor regulates two or more traits, and plasticity is favored in one trait but not the other [43].

### Wnt signaling regulates both wing shape and melanic patterning

To investigate the potential mechanism underlying wing shape and melanization patterning in *Oncopeltus*, we investigated the Wnt signaling pathway, which is involved in both wing growth and pigmentation patterning in several insect species, including butterflies [25–28, 30, 33, 34]. Because knocking down *wg* had no observable effects on the wings (data not shown), we investigated the effects of silencing *arm*, whose protein product is involved in mediating the canonical Wnt signaling pathway [29]. Two *arm* paralogs, *arm1* and *arm2*, were identified in the *Oncopeltus* genome, and we silenced each of these genes via RNAi to determine the effect of Wnt signaling disruption during wing development.

**Fig. 7:**
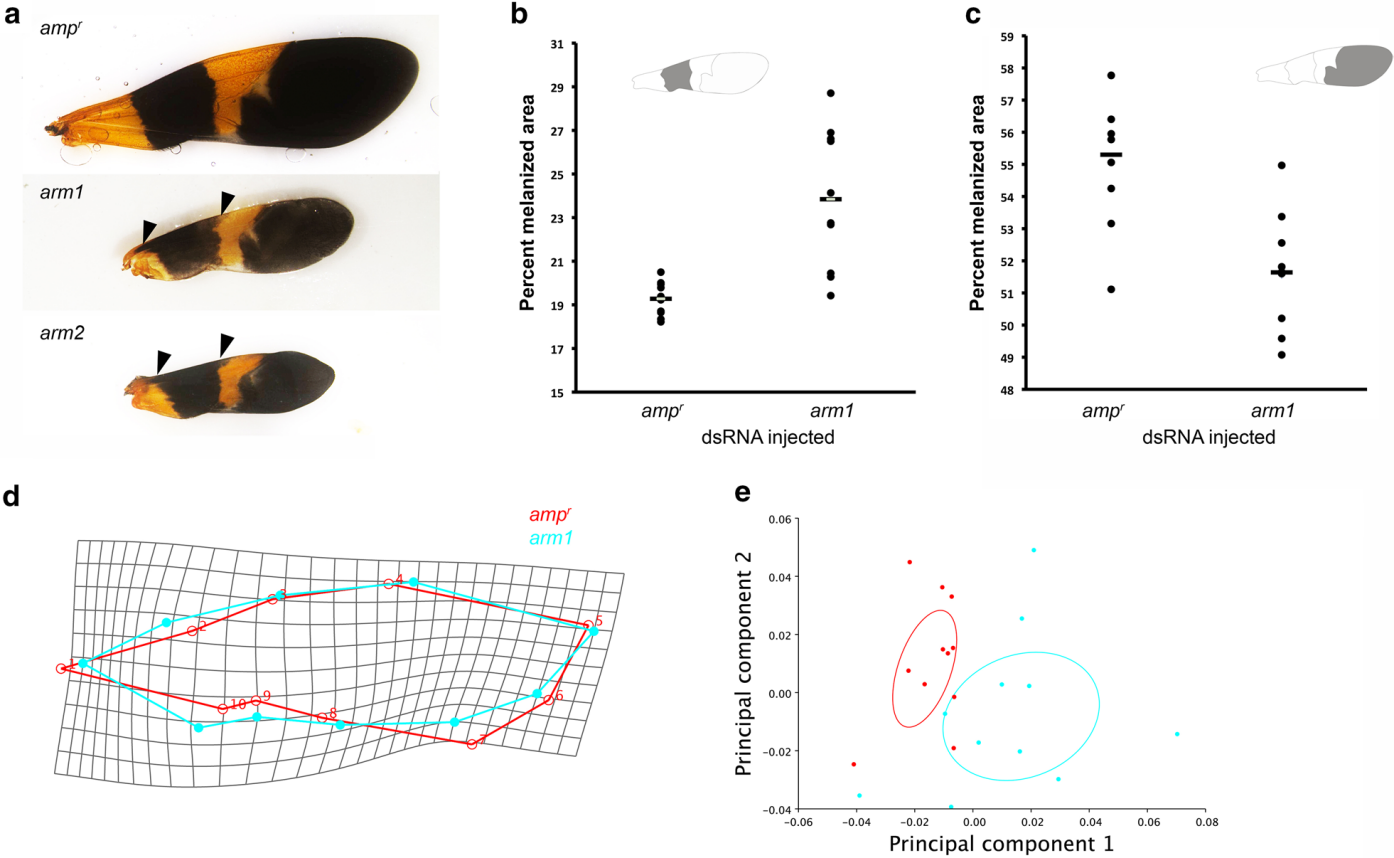
Effects of *arm1* and *arm2* knockdown on adult *Oncopeltus* wing pigmentation and shape. **a** Wings of *amp* ^*r*^ (control), *arm1* and *arm2* dsRNA-injected adults. The anterior portion of the proximal band is enlarged in *arm1* and *arm2* knockdown wings (*arrowheads*). Effects of *arm1* removal on the normalized areas of the proximal band (**b**) and the distal membranous portion (**c**) of the forewing. **d** Shape change caused by *arm1* knockdown as depicted by the wireframe diagrams for the *arm1* and *amp* ^*r*^ knockdown wings superimposed on a deformation grid. A scale factor of 3 was used. **e** Principal components analysis of *amp* ^*r*^ and *arm1* knockdown wings with 95 % confidence ellipses

Of the fourth instar nymphs injected with *arm1* dsRNA, 14 out of 21 survived to the adult stage and exhibited altered phenotypes. Knockdown of *arm1* led to alterations in forewing shape and pigmentation patterns (Fig. 7). In particular, the shape of the proximal black band was altered such that the anterior portion was expanded compared with the *amp*^*r*^ knockdown animals (Fig. 7a; black arrowheads). The area of this band was also increased relative to the area of the entire forewing (Fig. 7b). In contrast, the relative area of the distal melanized membranous portion of the wing decreased in size (Fig. 7c). In addition to altering the melanization, the wing shape was altered in *arm1* knockdown animals. Morphometric analysis showed that the width of wing was reduced at its most distal tip in *arm1* knockdowns compared with *amp*^*r*^ knockdown animals (Fig. 7d). Principle component analysis showed that the *arm1* and *amp*^*r*^ knockdown wings occupy distinct morphospaces (Fig. 7e). Finally, a discriminant function analysis was performed with MorphoJ, and the difference between the *arm1* and *amp*^*r*^ knockdown wings was significant [*t*(226.1914); *p* = 0.0257].

Injection of 10 ng to 1 µg *arm2* dsRNA was lethal to the nymphs. Although two out of four nymphs survived to the adult stage after being injected with 1 ng of *arm2* dsRNA, both exhibited wild-type phenotype. One out of eight nymphs survived to the adult stage when injected with 2 ng of *arm2* dsRNA. This adult had reduced wings and an altered proximal melanic band that resembled that of the *arm1* knockdown wings (Fig. 7a). These results suggest that Wnt signaling regulates both wing shape and melanin patterns and that the pleiotropic effects of Wnt signaling may constrain the amount of plasticity of melanic bands.

## Discussion

In this study, we sought to understand how developmental modules behave in response to environmental changes by studying melanization in *Oncopeltus fasciatus*. Consistent with a previous study by Novak [36], we found the abdominal pigmentation of *Oncopeltus fasciatus* to be sensitive to temperature. Additionally, we found that the reaction norms exhibit a continuous response to varying rearing temperatures and that the temperature-sensitive period occurs primarily during the fifth nymphal stage. Although *Abd*-*B* knockdown partially phenocopied the pigment patterns observed at lower rearing temperatures, temperature appears to act on a regulator downstream of Abd-B to influence melanization. In contrast, wing melanism of *Oncopeltus* was robust to temperature fluctuations. Silencing *arm1* or *arm2* altered both the wing shape and pigmentation patterning, indicating that the pleiotropic effects of Wnt signaling in the wing may constrain the amount of plasticity observed in the melanic elements. Thus, distinct upstream mechanisms control melanin production and their plastic response to temperature.

Plasticity in pigmentation in arthropods can arise from two separate mechanisms. The first type occurs after ecdysis into the adults and depends on the pigment granule movement. For example, in the grasshopper *Kosciuscola tristis*, temperature influences melanism by impacting the movement of the pigment granules in the adults [44]. In contrast, in other insects, adult pigmentation is determined earlier in development through alterations in the expression of pigmentation regulators [45, 46]. Our study indicates that the mechanism underlying *Oncopeltus* abdominal melanin plasticity is consistent with the latter type, whereby temperature-sensitive mechanisms determine the amount of adult cuticular melanism before the adult cuticle is laid down.

Pigmentation in many insects is regulated by a two-step process where patterning genes control the spatial and temporal specification of pigmentation and effector genes perform the actual biochemical synthesis of pigments [18]. The partial knockdown of the gene coding for the melanin synthesis enzyme *Ddc* did not phenocopy the effects of temperature on the pigmentation patterning, although overall melanization was reduced (Fig. 5), indicating that modulation of pigment biosynthesis alters the intensity of the pigment patterns but not the shape or the size of the pigment pattern. However, pigment biosynthesis genes may still regulate plasticity of pigmentation intensity even if they do not regulate the size and shape of the patterns. In *Daphnia*, for example, plasticity in melanism of the whole body has been shown to be controlled by distinct levels of *Ddc* [20]. We have also observed darker whole bodies in *Oncopeltus* reared at 20 °C compared with those raised at 33 °C (Fig. 1c, d), and such plasticity in whole-body pigmentation may also be controlled by changes in the pigment biosynthesis pathway. In contrast, changes in the shapes and size of melanic patterns likely depend on upstream regulators.

Knockdown of posterior Hox gene *Abd*-*B* led to partial phenocopy of the shape changes induced by lower rearing temperatures resulting in extra pigmentation in segment A5 that normally lacks pigmentation at 26.5 °C (Fig. 4). Nonetheless, even when *Abd*-*B* is knocked down, the melanic bands remain phenotypically plastic, indicating that temperature affects a mechanism downstream of Hox genes to influence phenotypic plasticity. In *Drosophila*, plasticity in abdominal melanization is also regulated by Abd-B and its influence on pigmentation genes as well as chromatin regulators [12] although it is not clear to what extent plasticity in shape and pigmentation intensity are uncoupled in this species.

**Fig. 8:**
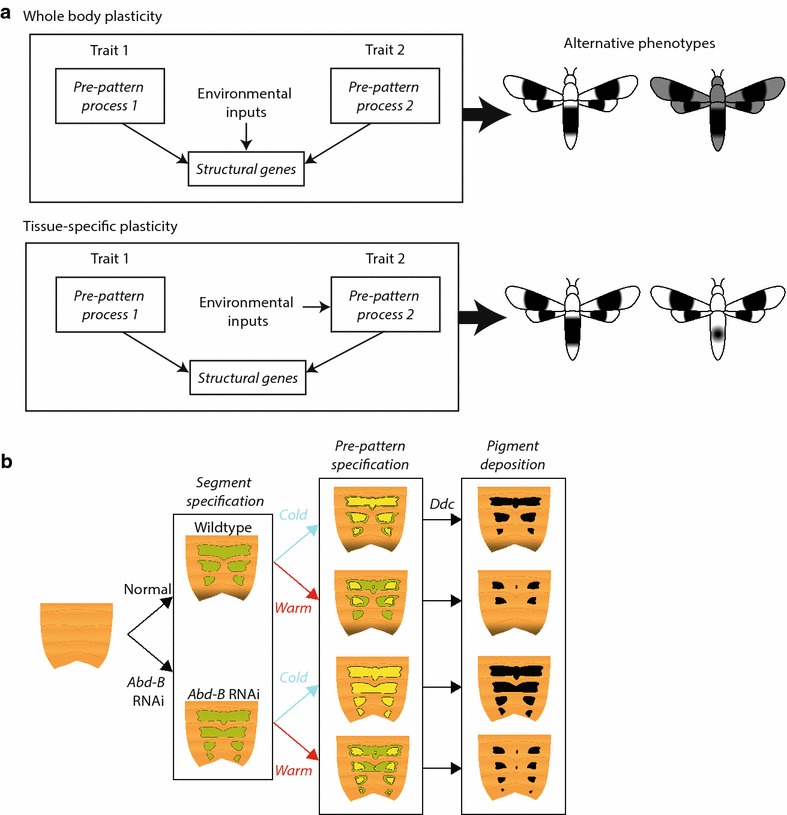
Models for **a** the regulation of whole-body and tissue-specific plasticity and **b** the abdominal melanization illustrating how temperature affects melanin production. **a** Environmental sensitivity in shared structural genes lead to whole-body phenotypic plasticity (*top panels*), while environmental sensitivity in tissue-specific pre-pattern mechanism leads to tissue-specific phenotypic plasticity (*bottom panels*). **b** In *Oncopeltus*, Hox genes specify the segments that can produce melanins. Subsequently, temperature affects the regulatory mechanism underlying specification of the size and shape of the abdominal melanic elements. Melanin biosynthesis enzymes, such as DDC, then produce melanin in these regions

Our findings suggest that temperature likely influences a component of the melanin patterning downstream of Abd-B but upstream of the melanin biosynthesis pathway. We propose that Abd-B acts to specify the pre-patterning of pigmentation by restricting the region that the pigment could potentially form. The size of the bands, however, is determined by regulators downstream of Abd-B. These regulators in turn would then activate the melanization enzymes to convert melanin precursors into melanin (Fig. 8). Many genetic loci have been linked to variation in abdominal pigmentation of *Drosophila* [23, 24]. Thus, it is possible that several processes are influenced by temperature to impact the final melanization of the abdomen. The broad sensitive period observed here is suggestive of a complex polygenic regulation of abdominal pigmentation plasticity.

In contrast, our study on wing melanism showed that wing size/shape is correlated with the positioning and size of the melanic bands. While wing size varies with temperature, the area of the melanic pigmentation scales with the wing size, leading to a relatively constant normalized area of the melanic region across various temperatures. We also showed that the modulation of Wnt signaling affects both the melanic bands and the wing size or shape. Wnt signaling has also been implicated in the regulation of the bands of the nymphalid ground plan symmetry system, which also tend to be more robust to environmental changes than the eyespots that develop independently of Wnt signaling [25–28, 47]. Thus, Wnt signaling, in addition to regulating wing shape [42], also plays a role in wing pigmentation patterning in several insect species. Pleiotropy is one of the genetic costs of plasticity [43], and the pleiotropic effects of Wnt signaling may pose a constraint on wing pigment plasticity. Given that wing shape and size have important fitness consequences [48–50], we propose that stabilizing selection acting on wings may constrain the amount of plasticity that an individual can exhibit. Further characterization of the precise regulation of wing patterning by Wnt signaling is necessary to elucidate potential links between wing shape and pigmentation.

Both modularity and plasticity have been theorized to promote morphological diversification [4, 51–53]. Our findings suggest that wing patterns are highly robust and are therefore likely to exhibit limited variability in nature. Thus, we predict that wing patterns would evolve more slowly, and in fact, closely related *Oncopeltus* species have similar wing patterns. In contrast, the combination of environmental sensitivity and modularity would be expected to promote rapid evolutionary changes and thus evolvability. Thus, plasticity in the patterning genes should promote rapid diversification of abdominal patterns although the adaptive significance of the abdominal pigmentation plasticity remains unknown.

## Conclusions

We are now beginning to understand how shapes and sizes of traits of organisms are regulated and are in the position to investigate how phenotypically plastic traits are regulated in a modular fashion. In some cases, the same homologous structure can exhibit distinct responses in the same organism due to heterogeneity in the local environment [54, 55]. Plants are particularly likely to exhibit such modular responses. In other cases, even if the structural genes are identical, traits can exhibit tissue-specific differences in plasticity because of distinct underlying developmental mechanisms. Our study suggests that plasticity in the pre-pattern specification is responsible for tissue-specific plasticity in the shape of melanic patterns of *Oncopeltus*. We propose that when plasticity arises within the structural genes shared by multiple traits, an organism’s phenotype can respond as a developmentally integrated system to environmental changes (Fig. 8a). In contrast, even though the downstream structural genes are the same, differential plasticity in the upstream regulation can lead to remarkably different tissue-specific modular responses to the environment even in the same organism. Recent studies have highlighted the importance of phenotypic plasticity in organismal evolution [51, 52, 56]. We speculate that the combination of modularity and plasticity should dramatically enhance evolvability of traits. In the future, it will be informative to investigate how tissue-specific developmental regulators of phenotypic plasticity evolve to generate novel phenotypes.

## Additional files

10.1186/s13227-016-0053-7 *Armadillo 1* and *armadillo 2* sequences cloned for silencing.
10.1186/s13227-016-0053-7 Knockdown verification for *arm1*, *arm2*, *abd*-*A*, *Abd*-*B* and *Ddc* using semiquantitative RT-PCR. Using *ribosomal protein subunit 3 (rps3)* as a loading control, expression was analyzed for day 2 fifth instars of *Oncopeltus* injected with control, *arm1* (1 µg), *arm2* (10 ng), *amp* ^*r*^ (1 µg), *abd*-*A* (0.1 µg), *Abd*-*B* (1 µg) and *Ddc* (1 µg) dsRNA during their fourth nymphal stage. The fourth instars for dsRNA injection were chosen at random and maintained at 26.5 °C.
10.1186/s13227-016-0053-7 Examples of ventral abdominal melanization of females (A) and males (B) transferred on various days of the fifth nymphal instars from 33 °C to 20 °C and vice versa.
10.1186/s13227-016-0053-7 Examples of ventral abdominal melanization of *Abd*-*A* knockdown animals.

## Abbreviations

abd-A: abdominal-A
Abd-B: abdominal-B
amp^r^: ampicillin resistance
arm1: armadillo 1
arm2: armadillo 2
DDC: dopa decarboxylase
dsRNA: double-stranded RNA
wg: wingless
