# Supplementary material for: The phenotypic plasticity of developmental modules
Source: EvoDevo. 2016 Aug 2;7:15. doi: 10.1186/s13227-016-0053-7 (PMC4971649; doi:10.1186/s13227-016-0053-7)
Supplement: Supplementary file 1 — 10.1186/s13227-016-0053-7 Armadillo 1 and armadillo 2 sequences cloned for silencing. [file 13227_2016_53_MOESM1_ESM.docx]

**Additional file 1**

***armadillo* sequences cloned for dsRNA synthesis**

*armadillo1*

AAGATGGTCTCCTTGCTTCAGCGAAACAACGTCAAGTTCCTGGCCATCGTGACTGACTGCCTTCAGATACTTGCTTACGGTAACCAAGAGAGCAAGCTGATCATACTCGCCTCGCAGGGGCCTATAGAACTCGTCAGGATCATGAGGAGTTACGACTATGAGAAACTTCTCTGGACTACTTCACGAGTTCTCAAAGTTCTGTCAGTGTGTTCGAGCAACAAACCAGCGATT

*armadillo2*

AGTAAAATGGCTGTGCGTGTAGCTGGGGGTCTTCAGAAGATGGTTCACCTCCTTCAGAGAAACAATGTAAAGTTCCTTGCAATAGTGACTGACTGTCTACAGATCCTTGCCTACGGTAATCAAGAGAGCAAGCTTATCATTCTTGCCTCTCAGGG
