# Supplementary figures and images for: The phenotypic plasticity of developmental modules
Source: EvoDevo. 2016 Aug 2;7:15. doi: 10.1186/s13227-016-0053-7 (PMC4971649; doi:10.1186/s13227-016-0053-7)

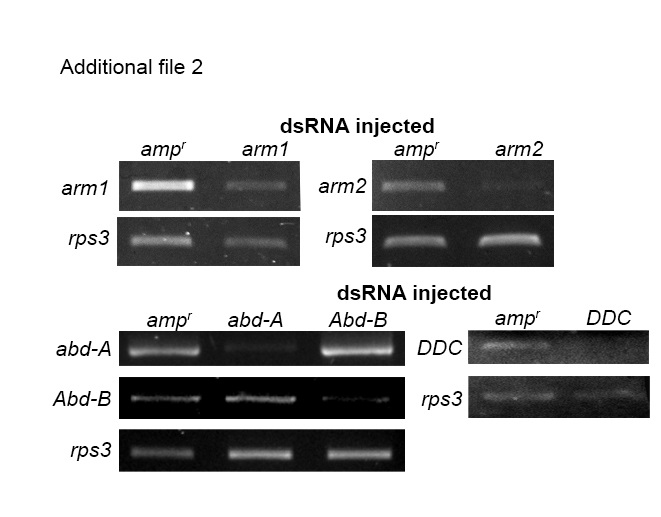

Supplement: Supplementary file 2 — 10.1186/s13227-016-0053-7 Knockdown verification for arm1, arm2, abd-A, Abd-B and Ddc using semiquantitative RT-PCR. Using ribosomal protein subunit 3 (rps3) as a loading control, expression was analyzed for day 2 fifth instars of Oncopeltus injected with control, arm1 (1 µg), arm2 (10 ng), amp r (1 µg), abd-A (0.1 µg), Abd-B (1 µg) and Ddc (1 µg) dsRNA during their fourth nymphal stage. The fourth instars for dsRNA injection were chosen at random and maintained at 26.5 °C. [file 13227_2016_53_MOESM2_ESM.jpg]

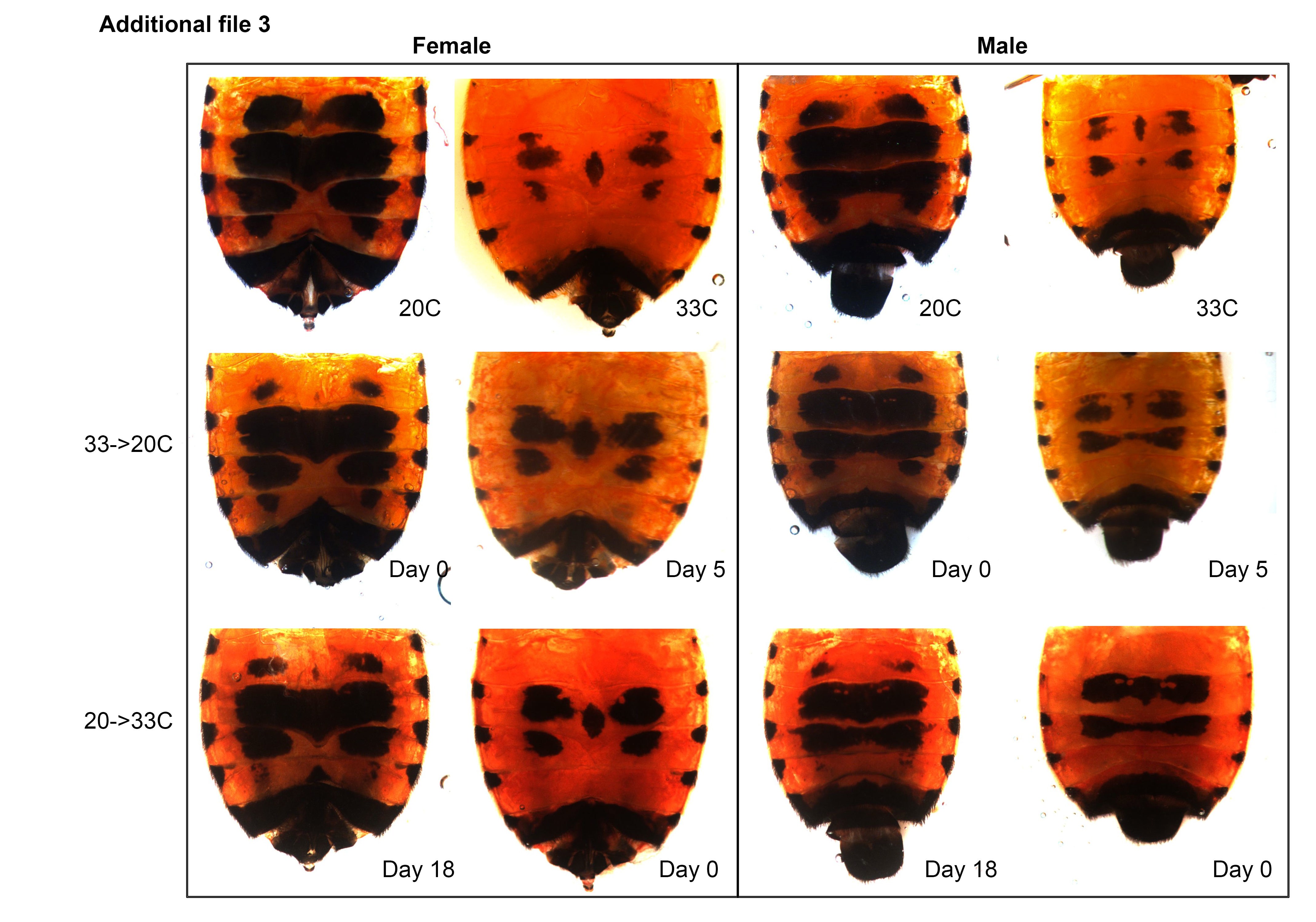

Supplement: Supplementary file 3 — 10.1186/s13227-016-0053-7 Examples of ventral abdominal melanization of females (A) and males (B) transferred on various days of the fifth nymphal instars from 33 °C to 20 °C and vice versa. [file 13227_2016_53_MOESM3_ESM.jpg]

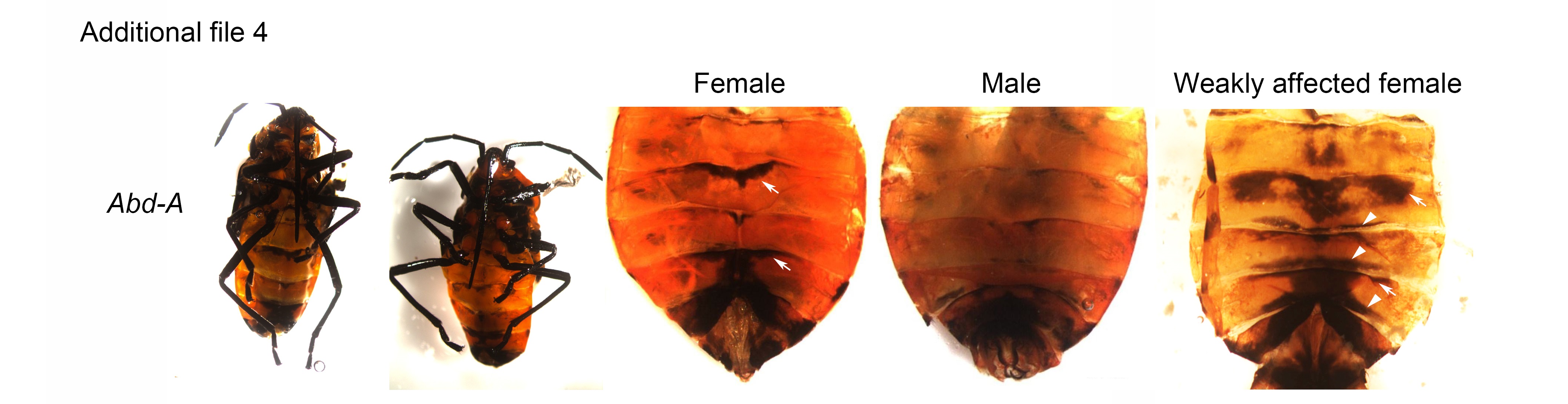

Supplement: Supplementary file 4 — 10.1186/s13227-016-0053-7 Examples of ventral abdominal melanization of Abd-A knockdown animals. [file 13227_2016_53_MOESM4_ESM.jpg]
